# Protocadherin-18 Is a Novel Differentiation Marker and an Inhibitory Signaling Receptor for CD8^+^ Effector Memory T Cells

**DOI:** 10.1371/journal.pone.0036101

**Published:** 2012-05-02

**Authors:** Edwin J. Vazquez-Cintron, Ngozi R. Monu, Jeremy C. Burns, Roy Blum, Gregory Chen, Peter Lopez, Jennifer Ma, Sasa Radoja, Alan B. Frey

**Affiliations:** 1 Department of Cell Biology, New York University Langone School of Medicine New York, New York, United States of America; 2 New York University Cancer Institute, New York University Langone School of Medicine New York, New York, United States of America; 3 Department of Pathology, New York University Langone School of Medicine New York, New York, United States of America; 4 Center for Cancer and Immunology Research, Sheikh Zayed Institute for Pediatric Surgical Innovation, Children's Research Institute, Children's National Medical Center, Washington, District of Columbia, United States of America; National Cancer Institute (INCA), Brazil

## Abstract

CD8^+^ tumor infiltrating T cells (TIL) lack effector-phase functions due to defective proximal TCR-mediated signaling previously shown to result from inactivation of p56^lck^ kinase. We identify a novel interacting partner for p56^lck^ in nonlytic TIL, Protocadherin-18 (‘pcdh18’), and show that pcdh18 is transcribed upon *in vitro* or *in vivo* activation of all CD8^+^ central memory T cells (CD44^+^CD62L^hi^CD127^+^) coincident with conversion into effector memory cells (CD44^+^CD62L^lo^CD127^+^). Expression of pcdh18 in primary CD8^+^ effector cells induces the phenotype of nonlytic TIL: defective proximal TCR signaling, cytokine secretion, and cytolysis, and enhanced AICD. pcdh18 contains a motif (centered at Y842) shared with src kinases (QGQYQP) that is required for the inhibitory phenotype. Thus, pcdh18 is a novel activation marker of CD8^+^ memory T cells that can function as an inhibitory signaling receptor and restrict the effector phase.

## Introduction

CD8^+^ CTL play an essential role in killing of virus-infected and transformed cells but in unmanipulated hosts fail to control tumor growth. Although the frequency of antigen-specific T cells in cancer patients is low, demonstrable priming occurs in response to tumor growth [1]. Investigation of animal models and tumor-bearing patients show production of antigen-specific CTL in the periphery but whose effector phase T cell function is suppressed upon entrance to the tumor [2], [3], a phenotype postulated to contribute to tumor escape from immune-mediated eradication [4]. This implies the tumor microenvironment induces TIL lytic dysfunction, a conclusion that was substantiated by several experimental approaches [5]. In a murine model of colorectal carcinoma (MCA38) nonlytic TIL were shown to be recently-activated effector memory cells (CD44^+^CD62L^lo^CD69^+^CD95L^+^CD122^+^CD127^+^ [6]). The dysfunctional lytic phenotype was subsequently shown to be due to a tumor-induced block in proximal TCR-mediated signaling that obviates ZAP70 activation, in turn due to rapid inactivation of p56^lck^ upon contact with cognate tumor cells [7]. During analysis of TIL p56^lck^ we observed that when nonlytic TIL form conjugates *ex vivo* with cognate tumor cells, p56^lck^ co-immuneprecipitates with a 120 kD protein, but whose identity and potential role in regulation of TIL function was unknown.

We have identified this novel p56^lck^ interacting partner: the adhesion molecule Protocadherin-18 (‘pcdh18’). We show that in cells of the hematopoietic lineage pcdh18 is expressed in activated central memory CD8^+^ T cells (CD44^hi^CD62L^hi^CD127^hi^) coincident with differentiation to the effector memory phenotype: CD8^+^CD44^+^CD62L^lo^CD127^hi^. pcdh18 is expressed in endogenous CD8^+^ memory cells that accumulate as mice age, or those elicited by prior immunization with various antigens. In addition, transfection of pcdh18 into primary CD8^+^ T cells (which do not express pcdh18) imparts the nonlytic TIL phenotype: defective proximal signaling, loss of effector phase functions, and AICD. Thus, these data reconcile prior observations concerning p56^lck^ activation status in TIL [5], [7] and identifies a novel activation marker of CD8^+^ effector memory T cells which can also function as a negative regulator of proximal TCR signaling and therein effector phase function.

## Results

### Identification of a p56^lck^ binding protein in TIL

**Figure 1:**
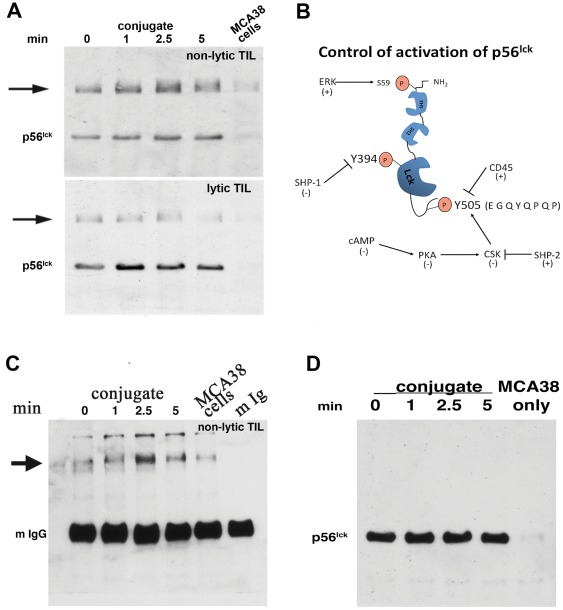
Reciprocal immunoblot analysis of p56^lck^ isolated from nonlytic and lytic MCA38 TIL. (1a) TIL were purified as described in [6] and either immediately used to form conjugates with MCA38 cells for the indicated times (upper panel) or plated in complete RPMI medium (lower panel) until performance of conjugation and reciprocal immunoblotting. Following incubation, detergent lysates were prepared and immuneprecipitated with Ab reactive with an epitope in the amino terminal portion of the protein (clone 3A5). Immuneprecipitated p56^lck^ was subjected to immunoblotting using anti-p56^lck^ (pY505) and detected by chemiluminescence following reaction with peroxidase-conjugated anti-rabbit. (1b) Schematic diagram of p56^lck^. The two primary sites of p56^lck^ regulation- the kinase activation motif (centered at Y394) and the inhibitory motif (centered at Y505) are indicated by boxes and key regulators of Y phosphorylation at each site are indicated. ‘+’ and ‘−’ indicate whether a given enzyme causes activation or inhibition of p56^lck^ activity. Activation of kinase function is mediated by phosphorylation of Y394 which is autophosphorylated upon dephosphorylation of Y505 (by CD45). Once phosphorylated, control of kinase function is mediated by Shp-1 dephosphorylation of Y394. Phosphorylation of Y505 (by Csk) prevents autophosphorylation of Y394 and Csk activity is controlled by cAMP regulation of PKA activity. The amino acid sequence of the Y505 motif is also indicated. (1c) Reciprocal immunoblot analysis of p56^lck^ using anti-pY Ab. TIL:MCA38 conjugates were formed for the indicated times as described above, extracts were prepared and immuneprecipitated with anti-p56^lck^ (Ab 3A5), and blotted with anti-pY (4G10). The position of mouse IgG (the species used for p56^lck^ immuneprecipitation, indicated as ‘mIgG’, and which obscures p56^lck^) is validated by analysis of purified mouse IgG in the last gel lane and the arrowhead indicates the interacting protein detected by anti-pY blotting. (1d) High mw band is not p56^lck^ dimer. Substitution of anti-pY505 with an anti-p56^lck^ reactive to a different p56^lck^ epitope than 3A5 (Ab 2102) was performed and detected only p56^lck^ migrating at the appropriate mw, and not a higher molecular weight species, eliminating the possibility that the higher mw band represented dimeric p56^lck^.

Analysis of p56^lck^ activation status in nonlytic TIL by immuneprecipitation and reciprocal immunoblotting using Ab reactive with the phosphorylated form of the src family kinase inhibitory motif (centered on Y505) showed that this motif in p56^lck^ was not appreciably phosphorylated upon conjugation *in vitro* with cognate tumor cells (Fig. 1a). However, a high molecular weight band (∼120 kD) co-immuneprecipitated with p56^lck^ and was recognized by motif-specific anti-pY505. The equivalent experiment using TIL that were briefly cultured *in vitro* before analysis (and therefore had re-established proximal TCR signaling and lytic function [5]), showed the presence of the 120 kD band but its abundance and conjugation-dependent phosphorylation was dramatically reduced compared to nonlytic TIL (Fig. 1a, lower panel). (Regulation of p56^lck^ centered on motifs Y394 and Y505 is shown diagrammatically in Fig. 1b). Since anti-peptide Ab may have significant non-specific crossreactivity, this analysis was repeated using anti-pY Ab (4G10) and produced equivalent results (Fig. 1c). A trivial possible basis for this observation (dimerization of p56^lck^ during cell lysis) was eliminated by reciprocal immunoblotting using a second Ab for blotting that is reactive with a different epitope of p56^lck^ which did not detect the ∼120 kD protein (Fig. 1d).

**Figure 2:**
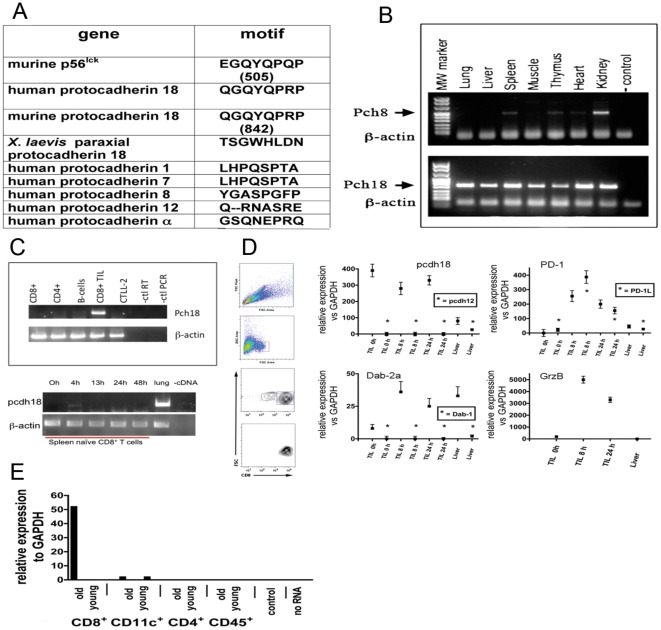
RT-PCR analysis of protocadherin 18 in tissues and immune cells. (2a) Amino acid sequence comparison of the p56^lck^ Y505 motif in protocadherins. (2b) RT-PCR analysis of mouse tissues. The indicated tissues and organs were isolated from a control mouse, RNA was extracted and used to prepare cDNA, and PCR performed using control (pcdh8 and β-actin) or pch18 primers as described in ‘Materials and Methods’. (2c) RT-PCR analysis of spleen cells. Spleens were isolated from a 7 week old mouse and the indicated cells were purified by FACS (top panel). TIL were isolated from an MCA38 tumor. CTLL-2 is a CD8^+^ lytic cell line. RNA was isolated and used to program RT-PCR as described in ‘Materials and Methods’. Similarly spleen CD8^+^ T cells were purified and activated *in vitro* with anti-CD3 for the indicated times before analysis (bottom panel). (2d) TIL qRT-PCR analyses. Single cell suspensions of MCA38 tumors were prepared and CD4^+^ or CD8^+^ TIL were isolated by magnetic immunobeading. (Liver tissue was isolated and used for RNA isolation in certain control analyses. TIL were labeled with anti-CD4 or CD8 Ab and further purified by FACS (example of flow cytometry analysis shown in left panels) before RNA isolation and qRT-PCR analysis. TIL used to prepare RNA immediately after isolation are indicated in as ‘nonlytic’ or ‘TIL 0 hr’. As indicated some TIL samples were cultured *in vitro* for 8 or 24 h before RNA isolation during which time TIL recover both proximal TCR-mediated signaling and lytic function [5], [7]. PCR analyses of various target RNAs are shown and include several control reactions that demonstrate specificity of the expression patterns observed (e.g. pcdh18 and pcdh12 in CD8^+^ TIL, Dab1 and Dab2a in CD8^+^ TIL, granzyme B in CD8^+^ TIL and liver, as well as TNF, IFN, IL-2, PD-1, and PD-1L). Data show SD from three independent experiments. (2e) qRT-PCR analysis of purified spleen cells. Spleen immune cells were isolated by FACS from young (4 week) or old (>48 week) control mice and RNA was isolated and used to program pcdh18 qRT-PCR as described in ‘Materials and Methods’. The data shown are representative of multiple repetitions.

These observations implied that a ∼120 kD protein: interacts with p56^lck^ in nonlytic TIL, contains the epitope recognize by anti-pY505, and is rapidly tyrosine phosphorylated upon contact with cognate tumor cells. BLAST analysis performed using as search query the p56^lck^ inhibitory motif peptide sequence to which anti-pY505 was raised (EGQYQPQP) identified a gene containing the sequence: QGQYQPRP; Protocadherin-18 (‘pcdh18’, [8]). Sequence comparison of protocadherin and related cadherin gene families revealed that pcdh18 is the only protocadherin member that contains a Y residue in the context of a Q/P motif (Fig. 2a) and also is the only non-src gene in the database to contain the src inhibitory motif. Expression of pcdh18 was examined by RT-PCR analysis of various tissues and analyzed closely-related pcdh genes for specificity control (pcdh8, Fig. 2b and pcdh12, Fig. 2d. qPCR primer sequences are listed in Table 1). pcdh18 RNA is widely expressed in adult tissues (and is expressed in cognate MCA38 tumor, dns), whereas expression of other pcdh genes is more restricted.

**Table 1:** Primers for qPCR.

GENE	5′→3′ SEQUENCE		qPCR Ta
**PD-1L**	FW	CCACGGAAATTGTCTGGTTG	60
**PD-1L**	RV	TGCTGCATAATCAGCTACGG	60
**PCDH-19**	FW	ACCATCACTTGTCTCCTCGGCTGT	58
**PCDH-19**	RV	CGTCCCGAGGTACAAGGCGG	58
**DAB-2b**	FW	CACTGATACCAGCCAATCCC	58
**DAB-2b**	RV	AATCAAGCTTGCAAGAGATTTGT	58
**DAB1**	RV	ACCAGCGCCAAGAAAGACT	58
**DAB1**	FW	ATCAGCTTGGCTTTGACCG	58
**PCDH-12**	FW	GTGAGGGGCAATGACAATCT	56
**PCDH-12**	RV	GAAGAGCTGTCGAGCCTGTT	56
**PD-1**	FW	CAGGCTGGGTAGAAGGTGAG	60
**PD-1**	RV	CATTCACTTGGGCTGTGCT	60
**IFN**	FW	AGCAACAGCAAGGCGAAAA	58
**IFN**	RV	CTGGACCTGTGGGTTGTTGA	58
**GRANZYME-B**	FW	CATGTAGGGTCGAGAGTGGG	58
**GRANZYME-B**	RV	CCTCCTGCTACTGCTGACCT	58
**IL-2**	FW	GTGCTCCTTGTCAACAGCG	58
**IL-2**	RV	GGGGAGTTTCAGGTTCCTGTA	58
**DAB2a**	RV	AAGTCATGCTGGCTCCACAG	58
**DAB2a**	FW	CACTGATACCAGCCAATCCC	58
**GAPDH**	FW	TGTGTCCGTCGTGGATCTGA	56
**GAPDH**	RV	CCTGCTTCACCACCTTCTTGA	56
**TNF**	FW	AGAGGCACTCCCCCAAAAGAT	58
**TNF**	RV	GCAGGAATGAGAAGAGGCTGA	58
**PCDH-18**	FW	TTTGGGGCGCGATTCTCCGA	56
**PCDH-18**	RV	GGACTCTGCACTCCTCCGTA	56
**PCDH-8**	FW	ATCTCGGGCCGAGAAGCTGA	56
**PCDH-8**	RV	CGGGGACACGTATCTGCTAC	56
**b-ACTIN**	FW	CCGTGAAAAGATGACCCAGATC	56
**b-ACTIN**	RV	CATACCCAGGAAGGAAGGCTG	56

(Ta = annealing temperature).

In order to identify the spleen cell type that expresses pcdh18, purified splenocytes from young control mice were analyzed by RT-PCR in comparison to nonlytic TIL and the CD8^+^ T cell line CTLL-2 (Fig. 2c). No signal was detected from major cell types including NK cells (even after activation when lytic function and IFN expression is high, Fig. S2. A modest PCR signal was detected in CD11c^+^ bone marrow-derived DC that increased upon activation, Fig. S1c). Cells isolable with the spleen capsule express pcdh18 (Fig. S1a) possibly accounting for the PCR signal obtained from total spleen preparations. To determine if pcdh18 expression was a function of activation status, CD8^+^ spleen cells from young naïve mice were treated with ConA but found to be negative (Fig. 2c). Nonlytic CD8^+^ TIL express pcdh18 RNA immediately upon isolation whose levels undulate with time of cell activation *in vitro* (Fig. 2d). Purified from the same tumor as CD8^+^ TIL, CD4^+^ TIL do not express pcdh18 (Fig. S2). Inhibitory signaling receptor PD-1 RNA is not detected in nonlytic TIL but is induced after brief culture *in vitro*, as is its ligand PD-1L.

We considered the possibility that the differentiation status of spleen cells may account for the inconsistent PCR findings and next compared FACS-purified CD4^+^, CD8^+^, CD11c^+^, and CD45^+^ cells from young (4 week) and aged (>48 weeks) control mice using qRT-PCR (Fig. 2e). CD8^+^ T cells of aged mice contain pcdh18 RNA and, since older mice contain a greater percentage of memory T cells compared to younger mice, prompted consideration that pcdh18 is expressed in CD8^+^ memory cells. Further supporting this notion is the previous observation that TIL are effector memory cells [6].

### pcdh18 is expressed in memory T cells

**Figure 3:**
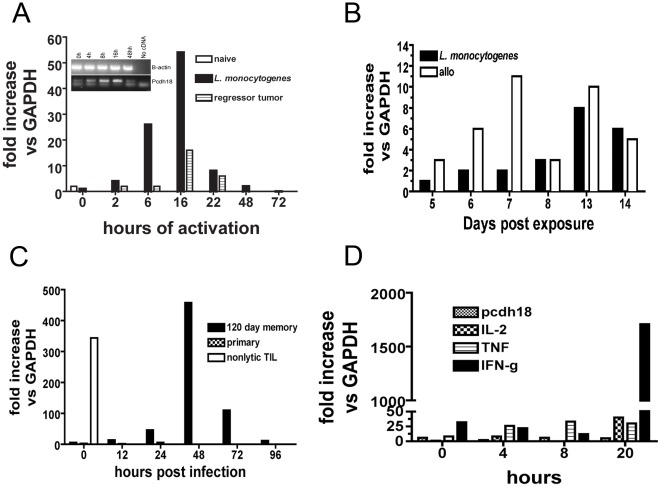
RNA analyses of spleen cells after induction of memory *in vivo*. (a) qRT-PCR analysis of spleen CD8^+^ T cells isolated from BL/6 mice previously infected with either 5,000 recombinant *Listeria monocytogenes*, buffer controls, or EL4 cells at a subtumorigenic dose (‘regressor tumor’). 28 days after exposure spleen CD8^+^ T cells were purified, activated *in vitro* with Con A for the indicated times, RNA prepared and qRT-PCR performed as described in ‘Materials and Methods’. (This analysis has been performed using mice previously infected for times up to 1 year with similar results). Insert shows gel analysis of pcdh18 RT-PCR expression from *L. monocytogenes*-immune mice. (3b) qRT-PCR analysis of purified CD8^+^ T cells from mice injected with *L. monocytogenes* or allogeneic H-2D spleen cells. (3c) qRT-PCR analysis of purified CD8^+^ T cells from mice originally infected with *L. monocytogenes* which were challenged by *in vivo* infection by *L. monocytogenes*. Spleens were isolated at the indicated times following challenge. Age-matched naive mice received only primary exposure given at the time of secondary challenge. Nonlytic TIL are shown for comparison. (3d) qRT-PCR analysis of purified control CD8^+^ spleen T cells and activated *in vitro* with anti-CD3e for the indicated times before RNA isolation and analysis by qRT-PCR. The data shown are representative of multiple repetitions.

A memory response was induced by either: infection with *Listeria monocytogenes* (and clearance), injection of allogeneic splenocytes (H-2D), or inoculation of a sub-tumorigenic dose of a transplantable syngeneic tumor (EL-4). At different times post antigen exposure (3 weeks up to >50 weeks) spleen total CD8^+^ T cells were isolated and pcdh18 expression analyzed. Expression of pcdh18 was low but in cells from immunized mice was rapidly and robustly increased upon *in vitro* activation (Fig. 3a). Induction started ∼2 h post activation and by ∼24 h expression was reduced close to that of non-activated cells. The rapid kinetics and transient nature of induction in cells following development of memory due to antigen exposure suggests pcdh18 is an immediate-early response gene of the memory response.

CD8^+^ T cells isolated following primary *in vivo* treatment express low levels (Fig. 3b) in comparison to re-activation of a memory response, approximately 6-fold less. Additionally, mice were infected with *L. monocytogenes* (or injected with allogeneic spleen cells, dns) to establish memory and subsequently challenged *in vivo* (Fig. 3c, pcdh18 expression in CD8^+^ TIL is shown at one time point for comparison). After priming by infection and resting before re-challenge (for more than four months in this case), pcdh18 was rapidly and robustly induced in total CD8^+^ splenocytes upon *in vivo* challenge showing that pcdh18 expression is not a phenomenon restricted to the *in vitro* experimental model. Control age-matched mice given only challenge (‘primary’) showed minimal pcdh18 expression reinforcing the notion that pcdh18 is expressed in memory CD8^+^ T cells. Transcriptional regulation of expression following *in vivo* re-activation, by either re-infection with *L. monocytogenes* (Fig. 3c) or re-injection of allogeneic cells (dns) demonstrates the physiological relevance of pcdh18 expression. (CD4^+^ T cells were also isolated from these groups of mice and did not express pcdh18 RNA, dns. Expression was never detected in CD4^+^ T cells, either TIL, naive or memory cells, Fig. 2c and Fig. S2). Expression of pcdh18 in CD8^+^ cells of naive mice following activation *in vitro* was assessed in comparison to selected cytokines- Fig. 3d). pcdh18 is not appreciably expressed in activated naive CD8^+^ T cells. Characteristic of naive cells, expression of effector phase cytokine RNAs is minimal until late in activation at which point IFN is robustly transcribed (Fig. 3d).

### Analysis of pcdh18 expression in memory cells

**Figure 4:**
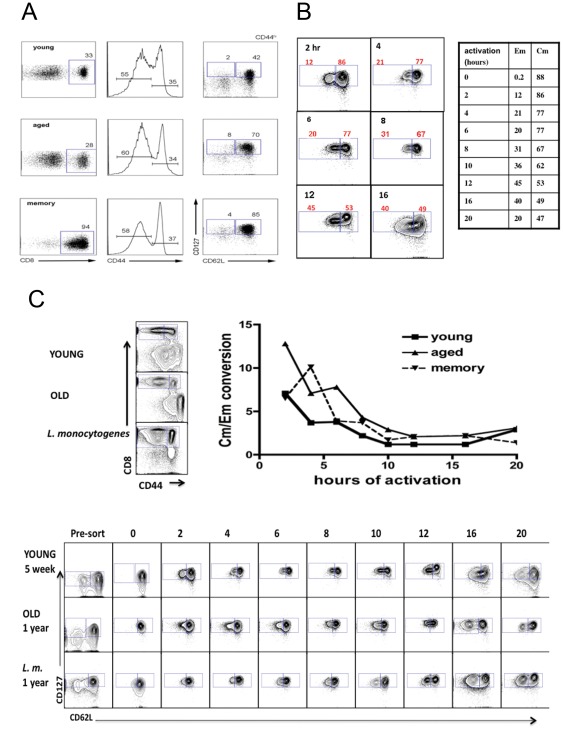
Conversion of Cm into Em cells upon activation *in vitro*. (4a) FACS purification of spleen CD8^+^ CD44^hi^CD62L^lo^CD127^hi^ (Effector-memory) and CD44^hi^CD62L^hi^CD127^hi^ (Central-memory) cells obtained from young (<5 week) and >50 week old naïve, and 50 week old mice that were previously injected with allogeneic spleen cells as indicated. Following sorting, an aliquot of cells was immediately taken for RNA isolation without activation. Analyses in the left panels are gated on CD8^+^ cells and show the distribution of CD44^+^ cells. Analyses in the right panels show Em and Cm cells within the CD44^lo^ and CD44^hi^ populations as indicated. (4b) Cm and Em cells were isolated by FACS from young, old, and memory (*L. monocytogenes*-infected) mice and activated *in vitro* with ConA for the indicated times before qRT-PCR. The % of CD8^+^CD44^hi^ cells as CD62L^lo^CD127^hi^ (Em) and CD62L^hi^CD127^hi^ (Cm from the ‘young’ cohort are shown in tabular form in this figure. Flow cytometry analyses of some samples from the kinetic experiment are shown to illustrate gating. (4c) Enriched CD8^+^ spleen cells prior to sorting are shown in the left panels. After labeling Cm were isolated and activated *in vitro*. The recovery of Em and Cm cells was determined by flow cytometry after re-staining and flow cytometric analysis. Shown are the FACS analyses after the indicated times of Cm activation. Also shown is the number of Cm and Em cells derived from *in vitro* activation of Cm cells represented as a ratio of Cm to Em cells at the various times of analyses.

In order to determine if pcdh18 is preferentially expressed in cells of a given differentiation state, CD8^+^ central memory T cells (‘Cm’, CD44^hi^CD62L^hi^CD127^+^) were FACS-purified from young (4 weeks), aged (ca 1 year), or mice that had been infected with *L. monocytogenes* >10 months prior (‘memory’). Central memory cells represented the major population of CD8^+^CD44^hi^ cells and effector memory cells were ∼4% of cells respectively in each group of mice (Fig. 4a). Preliminary experiments suggested that upon activation of purified Cm cells *in vitro*, pcdh18 was expressed starting at ∼2 h coincident with conversion to the effector memory phenotype (loss of CD62L). To extend those observations, following *in vitro* activation of endogenous Cm, Cm and Em cells were purified by FACS before RNA analysis by qRT-PCR (Fig. 4b+c). Selected FACS data for young mice is shown in Fig. 4b where a reciprocal relation is seen between decreased recovery of Cm cells and increased Em cells (as a percentage of live cells) as a function of time of activation. The number of Cm and Em cells recovered at various times after activation *in vitro* was plotted as a ratio of Cm to Em cells (‘Cm/Em conversion’, Fig. 4c). Aged mice had greater starting numbers of Cm cells, thus the ratio is initially higher for that group, but rate of loss of CD62L upon activation *in vitro* is very similar between the three groups. For all groups of mice the percentage of Cm cells declined steeply at early times of activation (Fig. 4c). Conversion of Cm (CD62L^hi^) to Em (CD62L^lo^) cells after *in vitro* activation likely reflects a combination of factors (including death) but primarily differentiation.

**Figure 5:**
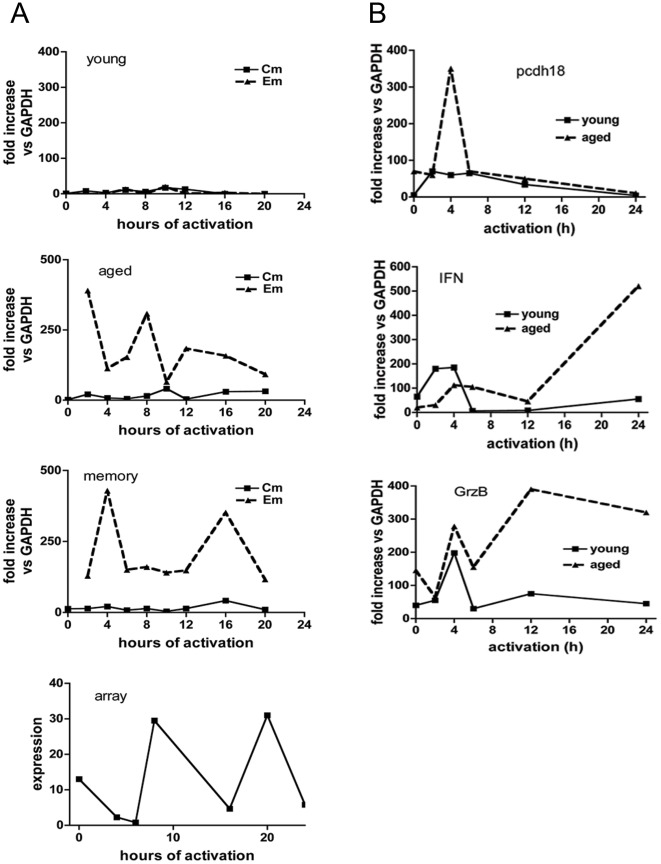
Protocadherin qRT-PCR and gene array analyses in activated Cm cells. (5a) pcdh18 qRT-PCR analysis of FACS-purified Cm and Em cells from young, aged, or ‘memory’ mice (from Fig. 4d) was as indicated (bottom panels). Gene array of FACS-purified Cm cells from a pool of 10 aged mice was performed by the NYU Center for Health Informatics and Bioinformatics. Purified Cm were activated *in vitro* and total cells in each culture were isolated for RNA extraction. cDNAs were hybridized to GeneChip arrays using an Affymatrix platform and the data were processed as described in Materials and Methods. (5b) Bone marrow CD8^+^ T cells were purified by positive selection magnetic immunobeading from young or aged mice, activated *in vitro* with ConA, and qRT-PCR performed as before.

pcdh18 qRT-PCR of FACS re-purified Cm and Em cells following *in vitro* activation of endogenous Cm cells is shown in Fig. 5a. Even after activation for extended time Cm cells from each model expressed comparatively low levels of pcdh18 RNA. In contrast, Em cells of aged and memory mice express robust levels even at the earliest activation time (2 h). The pattern of expression in Em cells was different between aged and memory mice and in aged mice levels undulated over the assay period with maxima approximately every 6 h. This temporal pattern reflects expression as determined by gene array analysis performed on a separate biological sample of Cm cells from aged mice (Fig. 5a, bottom panel). [In the array analysis Cm cells were activated *in vitro* but were not re-purified (to eliminate Em cells) before RNA isolation and thus contain CD62L^lo^ cells that differentiate *in vitro* under these conditions.]

The high level of expression of IFN, IL-2 and grzB in newly-differentiated Em cells from mice in which a memory response was deliberately induced (by *L. monocytogenes* infection, Fig. S3) supports the notion that CD44^hi^CD62L^lo^CD127^hi^ cells in immune (‘memory’) mice contain *bona fide* Em cells and young mice do not. Cm cells (CD62L^hi^CD127^hi^) show an obverse pattern of those cytokines associated with the effector phase. Collectively considered, pcdh18 is expressed relatively poorly in Cm cells and in Em cells the kinetics of expression closely follows the pattern of grzB, IFN, and IL-2 which is consistent with the notion that pcdh18 expression is restricted to recently activated Em cells.

In order to evaluate the expression of pcdh18 in a different population of memory cells total CD8^+^ T cells from bone marrow were analyzed (Fig. 5b). pcdh18 expression trended with paradigmatic RNAs of memory cells (IFN and grzB) in that expression was greater in old versus young mice supporting the generality of our findings.

### pcdh18 expression in primary CD8^+^ lytic T cells

**Figure 6:**
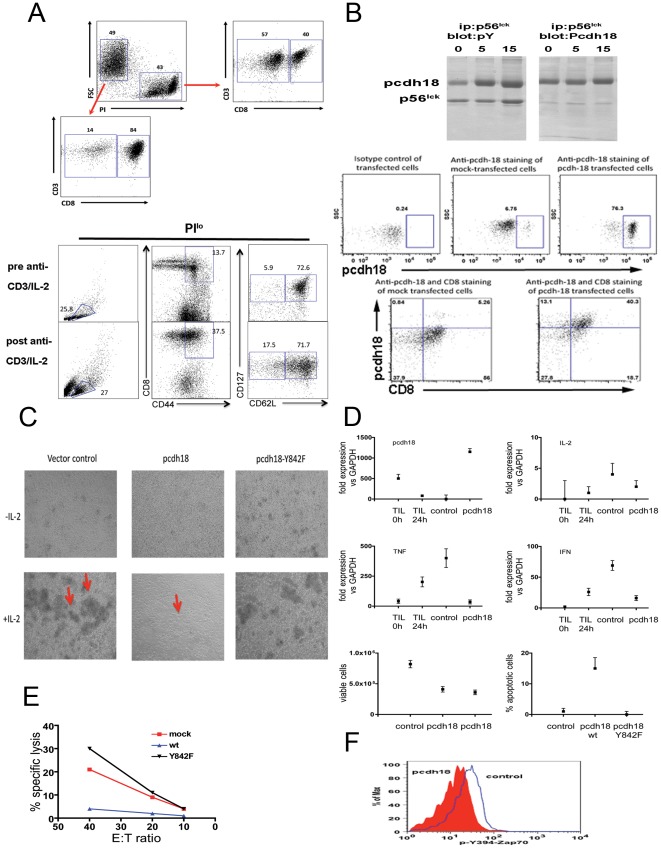
Biochemical and functional analyses of lytic T cells transfected with pcdh18. (6a) Flow cytometry analysis of primary lytic effector cells generated from spleens of 7 week old mice (prepared as described in ‘Materials and Methods’). PI^lo^ cells are >80% CD8^+^, PI^hi^ cells are ∼40% CD8^+^ (top panel). >70% of CD8^+^CD44^hi^ cells are CD62L^hi^CD127^hi^ (bottom panel). (6b) (top) Reciprocal immunoblot of p56^lck^ isolated from nonlytic TIL. Analysis was performed as described in Figure 1a (after conjugation with cognate MCA38 tumor cells for 0, 5, or 15 min as indicted and immune precipitated with anti-p56^lck^ Ab 2102- left panels- or Ab 3A5- right panels) and blots were probed with anti-pY or anti-Pcdh18 as indicated. (bottom) Expression of pcdh18 protein in transfected effector cells by flow cytometry was as described in ‘Materials and Methods’. Cells were stained with control or anti-pcdh18 Ab as indicated (top). (6c) Phase contrast microscopy of transfected cells. Effector cells were transfected as indicated and cultured *in vitro* in the presence or absence of IL-2 for ∼24 h before microscopy. Arrows indicate cell clusters. (6d) RNA was extracted from transfected effector cells (‘control’ or ‘pcdh18’), nonlytic TIL (‘TIL 0 h’), or lytic TIL that were activated with anti-CD3 for 4 hours (‘TIL 24 h’) and used for cytokine qRT-PCR, or cells were assessed for viability (PI and Annexin V staining), as described in ‘Materials and Methods’. In the viability assay two different plasmid constructs were used in separate experiments (bottom left panel) or the Y842F mutant (bottom right panel). (6e) Transfected effector cells were assayed for lytic function by re-directed cytolysis assay as described in ‘Materials and Methods’. (6f) Transfected cells were assayed for binding of anti-Zap70 pY493 after permeabilization following activation with anti-TCR for 2 min as described in ‘Material and Methods’.

Spleen cells activated *in vitro* with anti-CD3 (followed by IL-2 treatment) expand a population of CD8^+^CD44^hi^ cells coincident with development of lytic function (Fig. 6a) [9]. (Interestingly, these cells resemble memory cells in terms of being CD62L^hi^CD127^+^ and upon activation resemble Em cells in that they start to lose expression of CD62L). Since these cells do not contain pcdh18 RNA (Fig. 3d), in order to assess the effect of pcdh18 expression on effector phase function, pcdh18 was expressed in these cells. A cDNA encoding pcdh18 was obtained from a TIL library, sequenced and found to be in agreement with the published sequence (dns). An Ab raised to a recombinant cytoplasmic domain of pcdh18 used in reciprocal immunoblotting of TIL extracts (Fig. 6b top) confirmed the identity of pcdh18 (Fig. 1a). Expression of pcdh18 protein in transfected primary lytic effector cells was confirmed by flow cytometry analysis of where ∼32% of PI^−^CD8^+^ lytic effector cells are pcdh18^+^ (Fig. 6b).

Cells transfected with control vector form prominent clusters upon activation ∼24 h post transfection (Fig. 6c). The number and size of clusters in control plasmid transfected cells increased with culture time reflecting cell division. However, cells transfected with pcdh18 form dramatically fewer and smaller clusters implying an effect on activation. Expression of pcdh18 containing a point mutation in the src inhibitory domain homology (QGQYQP, Y842F) completely reverses the effect on cluster formation implying phosphorylation of the motif is important in pcdh18 function (see below). Similarly, expression of cytokine RNA in pcdh18-transfected cells was significantly diminished compared to controls (Fig. 6d). (Cytokine PCR analyses of nonlytic and lytic TIL is shown for comparison.) Further, analysis of AICD in pcdh18-expressing cells (bottom panels in Fig. 6d) showed a dramatic reduction in both cell recovery and viability: cells expressing pcdh18 are more Annexin V^+^ (early apoptosis, dns) and PI^+^/Annexin V^+^ (late apoptosis, Fig. 6d). (The induction of AICD by pcdh18 expression in primary T cells and high-density culture is reminiscent of PD-1 which upon ligation and TCR activation also causes AICD [10]–[12]). Similar to the effect on cluster formation, expression of the Y842F mutant significantly reverses enhanced AICD caused by wt pcdh18. In addition, more cells are recovered when transfected cells are not activated *in vitro* (dns) showing a requirement for activation in induction of cell death.

Expression of pch18 in primary effector cells also inhibited cytolysis (Fig. 6e), calcium flux (dns), and activation of Zap70 (Fig. 6f) confirming a site of action in the proximal TCR pathway. Similar to TIL or transfected primary CD8^+^ T cells (though less robustly) CD44^+^CD62L^+^pcdh18^+^ endogenous Cm cells in aged mice have diminished calcium flux and Zap70 activation compared to CD44^−^ naïve cells derived from the same mouse (Fig. S4b). Wildtype pcdh18 was shown to bind to p56^lck^ (Fig. 1) and phosphorylation of Y842 is required for inhibition of T cell functions (Fig. 6). Thus, pcdh18 is a strong candidate to mediate the effector phase defects in nonlytic TIL since each of these characteristic biochemical and functional deficiencies in transfected primary effector cells phenocopy nonlytic TIL [5]–[7], [13], [14].

## Discussion

The cell surface adhesion molecule pcdh18 is expressed widely in tissues but in the hematopoietic system primarily in CD8^+^ effector memory T cells (CD8^+^CD44^+^CD62L^lo^CD127^+^) derived from recently-activated central memory cells (CD8^+^CD44^+^CD62L^hi^CD127^+^). pcdh18 binds to p56^lck^ in activated primary CD8^+^ effector memory cells and is closely associated with defective: cytokine secretion, cytolysis, and blockade of proximal TCR signaling, and enhanced AICD; a phenotype that mimics that of nonlytic TIL [5], [7]. These effects are characteristic of inhibitory signaling receptors (ISR) expressed in NK cells and T cells that function to dampen cellular immune responses [11], [15]–[17].

p56^lck^ in CD8^+^ TIL interacts with pcdh18 coincident with robust phosphorylation at a tyrosine motif (Y842) shared with src kinases (QGQYQP). Y842 was shown by site-directed mutagenesis to be required for the pcdh18 inhibitory phenotype in transfected T cells. At early times during cell interaction the equivalent motif in p56^lck^ (Y505) is not phosphorylated [5]. Since Csk is known to phosphorylate p56^lck^ Y505, we hypothesize that it is responsible for the preferential phosphorylation of the homologous motif in pcdh18 (Figure S6). Supporting this notion is co-localization of Csk and p56^lck^ at the TIL immunological synapse [7] where, since pch18 and p56^lck^ interact (Fig. 1), it is reasonable to assume pcdh18 is also localized. The rapidity of inhibition of TCR signaling mediated by pcdh18 is characteristic of ISR expressed in immune cells, thus, within seconds of recognition of cognate tumor cell, p56^lck^ in anti-MCA38 TIL becomes inactive, therefore Zap70 is not activated and all downstream signaling is prevented which abrogates the effector phase. Although many studies have shown that a wide variety of inhibitory signaling receptors are expressed in cells of the adaptive immune response [16], [18], this is the first report of the adhesion molecule pcdh18 both being expressed in T cells and regulating proximal TCR signaling.

Since a role for pcdh18 in the immune system was not previously reported, by RT-PCR we determined its expression profile in CD8^+^ cells in different states of differentiation finding its expression is restricted to memory cells and is not unique to a particular experimental model including endogenous cells from aged naïve mice. The experiment in which Cm cells were purified and activated *in vitro* before purification of Em and Cm cells followed by RT-PCR analysis (Fig. 5) showed that neither Cm or Em originating from young mice robustly express pcdh18, in contrast to *in vitro* differentiated Em cells originating from aged mice. Perhaps there exists in young mice an endogenous CD44^hi^CD62L^hi^CD127^+^ Cm subpopulation that resists pcdh18 transcriptional activation under our *ex vivo* activation conditions. Alternatively, and more likely, memory cells identified by cell surface marker expression contain non-memory cells in addition to *bona fide* memory cells. We hypothesize that pcdh18 expression distinguishes the true memory population. In that experiment Cm cells from aged mice rapidly convert to Em cells (loss of CD62L) and the Em robustly express pcdh18 in an undulating kinetic pattern seen also by gene array analysis (Fig. 5a). The rapid kinetics of pcdh18 transcription induction *in vitro* or *in vivo* (Fig. 3c) further implies a role in memory re-activation.

That pcdh18 is a marker of Em formation is also supported by several observations including the robust expression in activated bone marrow-derived CD8^+^ T cells (Fig. 5b) and in isolated TIL. Further evidence that pcdh18 is a marker of authentic memory cells comes from the experiment in which TIL were adoptively transferred into RAG −/− mice (Fig. S4). Adoptive transfer of naïve cells into RAG −/− causes homeostatic expansion in which naïve cells acquire a cell surface phenotype that resembles central memory cells (CD8^+^CD44^+^CD62L^hi^CD127^+^) but these cells likely do not provide memory-mediated immune protection reflected by the lack of expression of IFN upon isolation. In contrast, IFN expression is extant (and able to be further induced) in cells derived from adoptive transfer of TIL, which do not express IFN in primary tumor tissue. pcdh18 transcription in adoptive transferred TIL and not in T cells derived from naïve cells (90 days post transfer into RAG −/−) after *in vitro* activation confirms that pcdh18 expression is exclusive to CD8^+^ T cells that have been previously activated, i.e. *bona fide* memory cells.

To define the signature of CD8^+^ Cm T cells during differentiation into Em cells, we profiled the gene expression patterns of purified Cm cells during the early stage of *in vitro* activation by microarray (Figure S5). Activation of Cm cells resulted in differential expression of many genes whose expression clustered into five groups each having distinct kinetics (Figure S5). The clusters containing the largest numbers of active genes had their maximal expression at 8 and 20 hours of activation and cluster 4 (having the third largest number of genes, and which contains pcdh18), has maxima at both 8 and 20 hours of activation. The profiles of Cm cells at each time point of activation (2–24 h) were compared to non-activated cells and the normalized expression array data of all 5,274 “active genes” (see ‘Materials and Methods’) are shown in Figure S6. Expression levels of selected genes from different clusters which have been validated (in Fig. S3) are shown in Figure S9.

The undulating kinetics of pcdh18 expression (peaking twice within 24 h) is shared by 972 genes (by cluster analysis, Fig. S5), which are nearly 20% of all active genes. This gene cluster (number 4) includes a candidate pcdh18-interacting partner Dab2 whose expression was also validated by RT-PCR. pcdh18 belongs to the cadherin superfamily: a large family of transmembrane glycoproteins that mediate calcium-dependent, homophilic cell-cell adhesion. Interestingly, the analysis revealed the expression of more than 250 additional glycoproteins in the same cluster. Among these glycoproteins are CD55, Masp1, Nfam1, IL12A, P2RX7, P2X, Thbs1 and TLR4, genes that are implicated in activation of immune response. In addition, nearly 25% of the genes that belong to this cluster are classified as “membrane” genes and are also likely to participate in cell activation. Notably, 31 genes of this cluster are classified as “cell cycle” genes including: Cdca2, Bub1, Cdc25c, E2f2, Cyclin D1, Fgfr2, and Fgf10 (and another 12 genes are classified as “M phase” genes). The concomitant expression of both pcdh18 – a mediator of TCR-signaling, and mitogenic genes that initiate cell cycle entry, suggests that pcdh18 may be influential in cell cycle entrance. Additional studies will reveal whether pcdh18 activation is cell cycle dependent. Furthermore, cell cycle entry of activated nonlytic TIL is typically followed by AICD [6]. Indeed, GO functional analysis of the early transcriptional response to Cm activation (cluster 2, genes that up-regulate 4 and 6 hours after activation) reveals strong enrichment of apoptotic genes in this cluster, among them the apoptotic facilitator BCL2-like 14, IFN, TNF and lymphotoxin A (Figure S7).

In Fig. S8 we also analyzed the array results for cluster distribution of ISR that have been shown to be expressed in T cells [16]. There are 8 ‘active’ ISR genes (defined as having the highest coefficient of variation; >15% of total) and 17 ‘non-active’ ISR genes expressed in activated Cm cells (this designation means that while expressed, the relative change in expression for this group is modest- having the lowest 85% of CV). Thus, 25 ISR genes are expressed during Cm cell conversion into Em cells which could potentially function to modulate proximal TCR signaling. This list includes several well-characterized ISR: PD-1, 2B4, CTLA-4, PEACAM (CD31), CEACAM-1 (CD66a), and CD85. RNA encoding a major ISR PD1 is expressed in freshly-isolated CD4^+^ TIL but not in CD8^+^ TIL (Fig. 2d). Upon activation of CD8^+^ TIL *in vitro* both PD-1 and its ligand PD-L1 are briskly upregulated with kinetics similar to GrzB. We interpret these data to mean that freshly-isolated TIL are nonlytic and express pcdh18 but not PD-1 RNA. In spite of upregulation of TIL PD-1 expression upon activation *in vitro*, if rested *in vitro* before activation, TIL regain lytic function diminishing a role for PD-1 in the nonlytic phenotype.

Gene array analysis showed that ‘exhausted’ CD8^+^ T cells that develop in response to persistent antigen load, are distinct from other T cells in different activation states, including anergic cells, and one gene prominently expressed in exhausted cells is PD-1 [19]. Since PD-1 RNA is not expressed in either nonlytic CD8^+^ TIL (Figure 2) and expression in Cm cells from aged mice is modest in comparison to Em cells (Fig. S3), exhausted cells appear to be distinct from both Cm cells and nonlytic TIL. Interestingly, upon activation PD-1 is transcribed in both cell types and with similar kinetics as several genes characteristic of effector cells (e.g. GrzB, IL-2, TNF) (Fig. S9) [11], [12]. A further distinction between nonlytic TIL and exhausted T cells is the rapidity with which TIL regain proximal TCR signaling and lytic function- within 2 h of purification [5]. However, exhausted T cells require dramatically longer to regain function (typically requiring PD-1 blockade [20]) and dysfunctional anergic T cells are thought to be very difficult to functionally recover.

As has been hypothesized previously, control of activation of the adaptive T cell immune response is tightly regulated by the activity of potentially a large number of ISR [16], [18]. Most ISR function as cell surface adaptor proteins that are themselves activated by an activating signal delivered to the cell (Ag recognition) and which function by recruiting an inhibitory phosphatase into proximity to its targets, often including the kinase responsible for activation of the ISR [17]. The rapidity and phenotype of inhibition of TCR signaling mediated by pcdh18 is characteristic of ISR expressed in immune cells which, although it differs from most ISR in lacking an ITIM motif and is activated by homophilic interaction in *trans* [21], functions equivalently (by binding directly to p56^lck^) in the inactivation of proximal TCR signaling. Like other ISR (e.g. PD-1), pcdh18 is transcriptionally regulated upon activation of memory cells. Thus, in Cm cells where pcdh18 RNA is relatively low, functional inhibition of TCR signaling can occur after gene expression.

Expression of several dozen ISR genes during activation of Cm raises the question how Cm cells can be efficiently activated? The fact that Cm cells are rapidly activated and expand *in vivo* upon re-exposure to antigen supports the notion that either: not all ISR transcripts are translated, that the encoded proteins are unable to function, or the inhibitory signal is superseded by the activation signal. It is also possible that ISR ligands are not expressed on APC during Cm re-activation thus Ag-dependent activation is unimpeded. The notion that multiple ISR are expressed upon T cell activation but the availability of any given ligand controls ISR activity is supported by the observations that dendritic cells and endothelial cells can express ligands for multiple ISR [22], [23] and tumors commonly express ISR ligands (e.g. MCA38 tumors express pcdh18, B7-1 [24] and possibly additional ISR ligands). Such redundancy in this system that restricts effector T cell function argues its physiological importance in governing the response to re-activation of the memory response.

Collectively, (as shown diagrammatically in Fig. S10) our observations suggest that upon activation of CD8^+^ memory cells, pcdh18 interacts with p56^lck^ and that the binding of p56^lck^ by pcdh18 is causal to the failure to activate ZAP70 and subsequent deficient effector phase function. Thus, we have identified a novel p56^lck^ binding protein that functions as an inhibitory signaling receptor during the effector phase in activated memory cells. pcdh18 is not only an inhibitory signaling receptor that can arrest the effector phase, but it is the first described marker that uniquely identify CD8^+^ T cells of memory origin.

## Materials and Methods

### Mice

C57BL/6 male mice were obtained from Jackson Laboratories (Bar Harbor, ME), were housed five per cage in a barrier facility, and were maintained on a 12 hr light/dark cycle (7 AM to 7 PM) with *ad libitum* access to food and water. A sentinel program revealed that tumor-bearing mice were Murine Hepatitis Virus-negative. Experiments involving animals were conducted with the approval of the New York University School of Medicine Committee on Animal Research.

### Tumors

MCA38 adenocarcinoma [5], [7] (a gift of Nick Restifo, National Cancer Institute) was passaged from tissue culture plasticware by incubation in HBSS containing 3 mM EDTA followed by washing in HBSS. Cell viability was determined by Trypan Blue dye exclusion and 1–2×10^5^ cells were injected intraperitoneally in a volume of 0.1 ml of HBSS for tumor induction. Cells were passaged *in vitro* for 3–5 weeks following which new frozen stocks were thawed for usage.

### Tissue culture

RPMI-1640 medium (Biowhittaker, Walkersville, MD) was used for growth of MCA-38 cells and for culture of T cells as described [6]. Thymoma EL-4 (ATCC) was passaged by dilution of media and CTLL-2 cells were maintained in media supplemented with rIL-2.

### Isolation of cells

Tumors were dissected, mechanically disrupted by passage through a tissue press, digested into single cell suspensions using collagenase, and TIL were isolated by immunomagnetic separation using type LS^+^ columns and anti-CD8a (or anti-CD4) conjugated magnetic beads (Miltenyi Biotec, Auburn, CA) as described previously [6]. Aliquots of isolated T cells were analyzed by flow cytometry and were routinely ∼95% CD8^+^. TIL were used immediately after isolation for experiments (‘non-lytic’) except in some experiments where TIL were plated in complete RPMI-1640 medium (∼2×10^6^ cells/ml) for 6–18 h before usage (‘lytic’). In some experiments TIL were further purified by FACS.

Splenocytes and bone marrow cells were usually enriched for CD8^+^ T cells by negative selection magnetic immunobeading (Miltenyi Biotec) followed by FACS. In some experiments splenocytes were labeled with anti-CD11c, B220, CD4 or CD8 and sorted using a MoFlo Legacy high-speed sorter. Cells were collected in media and immediately reanalyzed for purity in a LSR–II cytometer. Cells were collected and placed in Trizol immediately for RNA isolation and RT-PCR.

### cDNA cloning of pcdh-18

Primers were designed with restriction sites that are absent within Pcdh-18 for cloning into pIRES (SacI and XmaI). The primers used were, Pcdh-18-SacI-Forward: 5′ TTGAGCTCTGAGTGGCTGGAGGA, and Pcdh-18-XmaI-Reverse: 5′ TTCCCGGGACACCTCGGGATCTTC. A 28-cycle RT-PCR using cDNA generated from lung as a template and a high fidelity Taq (Phusion Taq, Life Technologies) generated a 3.4 Kbp product. The band was extracted from an agarose gel and purified using the ‘PCR clean up kit’ (Denville). pcdh-18-pIRES was then digested with SacI and XmaI, the products separated in an agarase gel, and the linear plasmid was extracted and purified. The plasmid was dephosphorylated and ligated to the PCR product (Ligation Kit, Life Technology) and used to transform DH5a. A selected clone was subjected to restriction digestion to confirm the presence of pcdh-18 which was also confirmed by DNA sequencing.

### Generation of a pcdh-18 point mutant (Y842F)

PCR primers were selected with restriction sites that are present within pcdh-18, KpnI (cDNA nucleotide 2287) and SspI (cDNA nucleotide 2557). The primers used were, pcdh-18-Y842-Forward: 5′ CACCAGGGGCAAT**T**TCAGCCACGGCCA, and pcdh-18-Y842-Reverse: 5′ AAGCATGGAGAGAAGCTGCGAGACCTC. The forward primer contains the mutated nucleotide (in bold T instead of an A) that generated the single point mutation in the product. A 30-cycle PCR using pcdh-18-pIRES as a template and a high fidelity Taq polymerase (Phusion Taq, Life Technologies) generated a 270 bp product. The band was extracted from an agarose gel and purified using ‘PCR clean up kit’ (Denville). pcdh-18-pIRES was digested with KpnI and SspI. The digested products were separated in an agarose gel and the linear plasmid was extracted and purified. The plasmid was dephosphorylated and ligated to the PCR product (Ligation Kit, Life Technology) and used to transform DH5a. A selected clone was subjected to restriction digestion to confirm the presence of pcdh-18 and confirmed by sequencing.

### Transfection of primary CD8^+^ T cells

Splenocytes were isolated, plated in complete media at 5×10^6^ cells supplemented with 10% of conditioned media from 2C11 hybridoma. After 36 h cells were collected and re-plated at 2×10^6^ cells/well supplemented with 10% of conditioned medium from an IL-2 producer line. 0.008 mg of plasmids was used to transfect by nucleofection (using program X-001, Lonza). Cells were cultured for 4 h in complete media containing IL-2. For signaling experiments, cells were then cultured in RPMI medium lacking IL-2 and FBS for 3–4 h prior to any experiment.

### Gene Array sample preparation

Ten spleens were pooled from 62 week old C57BL/6 male mice and enriched for CD8^+^ T cells by negative selection using a cocktail of biotinylated antibodies to deplete CD4, CD11c, CD11b, MHC-II, B220, and NK cells. Cells were then stained for CD8, CD44, CD62L, and CD127 and sorted (using a iCyt Reflection parallel cell sorter). Cells were collected and cultured (0.5×10^6^ cells/well) in 10% complete RPMI media supplemented with 0.005 ugr Con A and RNA prepared using TRIZOL and the ‘RNeasy clean-up kit’ (Invitrogen) after activation for different times.

We established transcriptional profiles for 6 time points after T cell activation (4, 6, 8, 16, 20, 24 h) including a zero h time point (prior to activation). RNA was isolated by standard procedures and its quality was assessed by the NYU Center for Health Informatics and Bioinformatics. cDNAs were hybridized to GeneSpring arrays using the mouse genome MOE430 2.0 array (Affymetrix) which interrogates ∼45,000 transcripts. The data discussed in this publication have been deposited in NCBI's Gene Expression Omnibus and are accessible through GEO Series accession number GSE34618.

### Analysis of gene expression data

Utilizing GeneSpring 7.2 (Agilent), raw Affymetrix CEL files were processed and normalized by applying the Robust Multi-Array Average expression measure (RMA) and baseline scaling. These metrics were further filtered to obtain 35,160 “*valid genes*”, representing transcripts (gene probes) that were detected as “Present” in at least one sample across all tested time points. To obtain a subset of variable genes, we calculated the coefficient of variation (CV) for each transcript and generated a set of 5274 “*active genes*” containing the transcripts with the highest (15% of the total) CV scores. For discovering prominent expression patterns we used the EXPANDER program [25] and executed CLICK, a novel clustering algorithm [26] that makes no prior assumptions on the structure or the number of the clusters. CLICK discovered five unique expression patterns. Cluster 4 has a unique undulating kinetics and includes pcdh18 that peaks twice within 24 h. We utilized functional annotations of murine genes provided by the Murine Genome Informatics, which uses the standard vocabulary introduced by the Gene Ontology (GO) consortium. Enriched functional categories (*p*≤0.01, after correction for multiple testing) were identified in each of the gene sets using EXPANDER, in which hypergeometric calculation is used to determine over-represented GO functional categories in a target set relative to a background set (the entire collection of putative murine genes) [27]. To avoid biases, genes represented by multiple probe sets were counted only once.

### Quantitative RT-PCR analysis

Total RNA was isolated (Trizol), converted into cDNA using Superscript II reverse transcriptase (Invitrogen, Carlsbad, CA), and was analyzed by real-time PCR (‘SYBR Green PCR master mix’, Applied Biosystems, Carlsbad, CA) using 200 fmol/uL primer concentration for 40 cycles. SYBR incorporation into PCR products was monitored using a model 7500 real-time PCR system (AB). Dissociation curve analysis (SDS 2.0 software) assessed the specificity and integrity of the PCR products. Degradation of total RNA used in the cDNA synthesis was normalized by determining the threshold cycle (Ct) values for target genes and housekeeping genes (b-actin and GAPDH) in each sample. Target gene/housekeeping gene ratios were calculated as: ratio = 2^[Ct(housekeeping)−Ct(target)]^. Relative mRNA levels are expressed as ‘fold induction’ for samples at different times of activation with ConA compared to time ‘0’ (ca 2–72 hr). PCR reactions were conducted in triplicate and repeated at least once with unique samples.

Primers used were: Pcdh18, Pcdh12, Pcdh8, IL-2, TNF, IFN, b-actin, GAPDH, granzyme B, PD-1, PD1-L, Dab1, Dab2a, Dab2b (sequences provided in Supplement).

### Antibodies

Antibodies, reagents, and procedures used in immunoblotting and flow cytometry were as described previously [5]. Additional Ab used were: phospho-p56^lck^ (Ab Y505/527, Cell Signaling Technology, Beverly, MA), phosphotyrosine (clone 4G10, Cell Signaling Technology, Beverly, MA), p56^lck^ (mouse Ab 3A5; Santa Cruz Biotechnology), p56^lck^ (Ab 2102; Santa Cruz Biotechnology), rabbit anti-Pcdh18 was prepared by creation of a GsT fusion protein containing the cytoplasmic domain of Pcdh18 and hyperimmunization of rabbits. Rabbit anti-pcdh18 with a similar specificity was also purchased (HPA017976, Sigma Chemical Company, St. Louis, MO).

### Flow cytometric analysis

All analyses were performed in general as described previously [5]. Primary Ab were added to cells (10^6^/mL) at empirically determined optimal concentrations. After incubation at 4° for 20 min, cells were washed once with 1 ml of FACS wash (PBS, 2% FBS) and fixed with 1% paraformaldehyde before analysis. For analysis of intracellular molecules, cells were first fixed and permeabilized using BD cytofix/Cytoperm (BD-bioscience). When used, secondary antibodies were diluted in the perm-wash buffer. Species-matched, control primary Ab was used to determine parameters and settings for flow cytometry.

### T cell activation

TIL (or activated primary splenocytes 4 h after transfection) were isolated and incubated in serum-free RPMI1640 medium for 3 h, washed, resuspended in cold compete medium, and anti-CD3e added for 30 min. Samples were collected and resuspended in warm medium containing anti-hamster Ig for 1, 2, or 5 min before preparation of detergent extracts or fixation for flow cytometry for staining with activation-specific anti-Zap70.

### Calcium flux

After transfection of primary spleen cells as described above, cells were incubated in 1%FBS RPMI1640 containing with 0.001 mg/mL biotinylated anti-CD3, 0.001 mg/mL biotinylated anti-CD8b, anti-CD8 PE, 0.0032 mg/mL Indo-1 AM (eBioscience) and 2 mM Probenecid (Sigma) for 30 minutes at RT° in the dark. Samples were washed twice with serum-free RPMI, then resuspended at 1×10^7^ cells mL. Cells were equilibrated to 37° five min before reading on a LSRII and were activated by crosslinking with 0.0125 mg/mL of streptavidin (Pierce).

### *Listeria monocytogenes*

*Listeria monocytogenes* (wt and recombinant expressing ova, a gift of Eric Pamer, MSKCI) were grown in BHK media and diluted in PBS for mouse injection (iv). A dose of 5–10×10^3^ bacteria was sufficient to induce infection in wt mice which was cleared in 3–4 days as assessed by colony formation on agar plate of extracts of various organs.

### Chromium-release assay

Cytolysis activity of TIL or transfected primary lytic effector cells was assessed in standard re-directed [^51^Cr]-release assays using P815 cells performed in quadruplicate wells for each E∶T ratio exactly as described [14]. Maximal release from target cells was determined by treatment of cells with 1% Triton X-100, spontaneous release was determined from cultures of labeled target cells incubated with medium only, and the formula used for determination of specific lysis was: (experimental release - spontaneous release)/(maximal release - spontaneous release)×100.

## Supporting Information

Figure S1 (a) pcdh18 PCR of fractionated spleen, related to Figure 2. Spleens from aged mice were used for analysis of pcdh18 expression. Single cells were prepared by mechanical disruption by grinding between the ends of frosted glass microscope slides in PBS followed by collection of fibrous material by settling at 1× g for 2 minutes. Cells in suspension were recovered by centrifugation, washed in PBS, and rbc lysed in hypotonic solution. Samples for analyses were: the non-manipulated organ (‘whole spleen’), fibrous material that was recovered by settling of the initial cell suspension at 1× g (‘capsule enriched’), cells taken immediately after mechanical disruption of spleen tissue into single cell suspension (‘disrupted spleen’), and cells of the single cell suspension following removal of the ‘capsule enriched’ fraction and lysis of erythrocytes (‘disrupted spleen (rbc lysed)’). (b) qRT-PCR and pcdh18 qRT-PCR primer efficiency. Spleen or bone marrow cells were harvested, enriched for either CD4^+^ or CD8^+^ T cells by negative selection as described above followed by staining with CD8, CD44, CD62L, and CD127 Ab. In some experiments other cell types (fractionated spleen preparations, CD4^+^ or CD8^+^ TIL isolated from MCA 38 tumors, total spleen CD8^+^ T cells, bone marrow-derived dendritic cells, or spleen NK cells) were analyzed after magnetic immunobead and/or FACS purification. (c) pcdh18 is induced by LPS treatment of bone marrow-derived DC. DC were prepared from bone marrow of C57BL/6 mice by standard methods [28] and either activated by overnight treatment with LPS or not before RNA isolation and analysis.
(TIF)

Figure S2 **TIL qRT-PCR analyses, related to** **Figure 2** **.** Single cell suspensions of MCA38 tumors were prepared and CD4^+^ or CD8^+^ TIL were isolated by magnetic immunobeading. All data are from freshly-isolated, nonlytic TIL. NK cells were isolated by magnetic immunobead positive selection from spleens of control or mice treated 24 h prior with Poly I:C as indicated. TIL were labeled with anti-CD4 or CD8 Ab and further purified by FACS (example of flow cytometry analysis shown in Figure 2) before RNA isolation and qRT-PCR analysis. Data shown are from a single experiment of two.
(TIF)

Figure S3 **qRT-PCR analyses of FACS-purified Cm and Em cells from young, aged, or ‘memory’ mice, related to** **Figure 3** **.** Central memory cells were isolated by FACS from young (5 week), old (1 year), or memory (infected with *Listeria monocytogenes* at 7–8 weeks of age and recovery until 1 year old) mice. Cells were activated *in vitro* for the indicated times and then purified by FACS into CD62L^hi^ (‘Cm’) or CD62L^lo^ (‘Em’) cell populations which provided RNA for qRT-PCR analyses.
(TIF)

Figure S4 (a) AT of TIL into RAG mice, related to Figure 4. CD8^+^ T cells were isolated by FACS from either pooled seven week old control mice spleens or as TIL from 7 week old mice bearing MCA38 tumors for two weeks. 2×10^6^ purified cells were adoptively transferred to RAG−/− mice (n = 6). Three months after transfer, spleen CD8^+^ T cells were isolated by magnetic immunobeading (∼2×10^6^ cells per mouse) and analyzed by RT-PCR before or after activation *in vitro* (0.005 mg/mL ConA for 20 h) as indicated. (b) Analysis of proximal TCR signaling in CD8^+^ Cm and naive effector cells of aged mice. Spleen cells were isolated from an aged control mouse, activated with either anti-CD3/CD8 crosslinking for different times (for calcium flux) or anti-CD3e (for Zap70 analysis), then assayed for activation as described in ‘Methods’.
(TIF)

Figure S5 **Microarray analysis of Cm cells, related to** **Figure 5** **.** Purified Cm cells were activated *in vitro* with Con A for the indicated times before cells were taken for RNA isolation and gene array analysis as described in ‘Material and Methods’. To obtain a subset of variably expressed genes, we calculated the coefficient of variation (CV) for each of the “valid genes” (see Material and Methods) and generated a set of 5274 “active genes” containing the transcripts with the highest (15% of the total) CV scores. For discovering prominent expression patterns we used the EXPANDER program [25] and executed CLICK, a novel clustering algorithm [26] that makes no prior assumptions on the structure or the number of the clusters. CLICK discovered five unique expression patterns.
(TIF)

Figure S6 **The normalized expression array data of all “active genes” are shown.** For each time of activation values for the probe-set signal and the assigned detection (Present/Marginal/Absent) are given. The “active genes” were further sorted in each cluster based on their CV score to present the most variable genes at the very top of each grouping.
(XLS)

Figure S7 **Presented is the David GO functional analysis.** Genes were classified into functional groups using the GO annotation tool [29] and overabundance was calculated using EASE software [29]. Functional classification of genes with an EASE score lower than 0.05 were marked as overabundant and included in the figure.
(XLS)

Figure S8 **Expression in activated Cm cells of ISR genes known to be expressed in T cells (** [**16]**, [17****] **was analyzed.** The data is presented in the top part of the figure for ISR that are “active” (having variable expression patterns- Fig. 5) and “non-active” genes (with relatively constant expression patterns) are shown in the bottom part of the figure. (Only transcripts with the highest 15% of the total CV sores were assigned into expression pattern clusters, thus ISR that having <85% CV have no cluster assignment).
(XLS)

Figure S9 **Gene array expression data for selected genes expressed in Cm cells upon activation.** Relative expression at different times of activation of selected genes for which PCR validation is available (In figure S3) is shown.
(TIF)

Figure S10 **Schematic of p56^lck^ regulation involving pcdh18, related to** **Figure 1** **.** This updated model differs from that shown in Figure1b in accommodating our data that shows that p56^lck^ Y505 is not phosphorylated upon activation of TIL and, likely also in memory CD8^+^ T cells since Zap70 is not activated in primary CD8^+^ T cells transfected to express pcdh18 [5]. In this model pcdh18 homophilic interactions between TIL and cognate MCA38 tumor disrupt TCR mediated signaling by binding to p56^lck^. Binding of p56^lck^ to pcdh18 causes a conformational change that prevents Csk (which localizes at the immune synapse with p56^lck^ [7]) from phosphorylating the p56^lck^ Y505 motif and thus permits phosphorylation of the homologous motif in pcdh18 (Y842). p56^lck^-Y384 is then available for targeting by Shp-1 causing p56^lck^ to deactivate as observed [5]. Thus, inactivation of p56^lck^ results in the inability to activate ZAP70, in turn preventing propagation of proximal signaling and loss of effector phase lytic function. The second diagram shows pcdh18 expressed in nonlytic TIL (or Em cells) engaged in homophilic interaction with pcdh18 expressed in a target cell; either tumor, endothelial cell, or potentially an activated DC. We propose the testable hypothesis: as a consequence of homophilic binding, recruitment of T cell pcdh 18 into proximity with Csk permits phosphorylation at Y842. Y842 phosphorylation in turn either permits or enhances binding to p56^lck^ leading to inactivation of kinase function or sequestration from its cognate targets TCRz or Zap70.
(TIF)
