# Supplementary figures and images for: Protocadherin-18 Is a Novel Differentiation Marker and an Inhibitory Signaling Receptor for CD8+ Effector Memory T Cells
Source: PLoS One. 2012 May 2;7(5):e36101. doi: 10.1371/journal.pone.0036101 (PMC3342238; doi:10.1371/journal.pone.0036101)

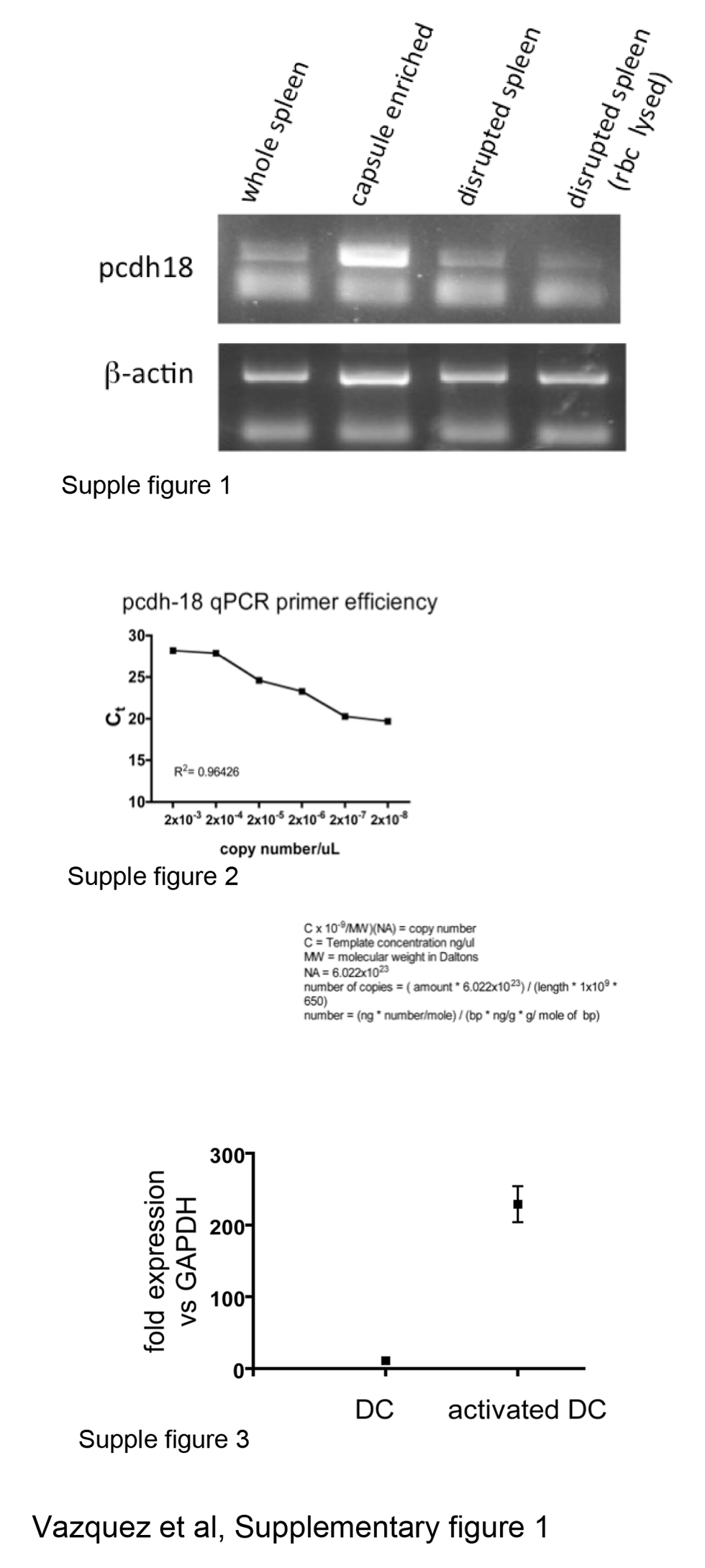

Supplement: Figure S1 — (a) pcdh18 PCR of fractionated spleen, related to Figure 2. Spleens from aged mice were used for analysis of pcdh18 expression. Single cells were prepared by mechanical disruption by grinding between the ends of frosted glass microscope slides in PBS followed by collection of fibrous material by settling at 1× g for 2 minutes. Cells in suspension were recovered by centrifugation, washed in PBS, and rbc lysed in hypotonic solution. Samples for analyses were: the non-manipulated organ (‘whole spleen’), fibrous material that was recovered by settling of the initial cell suspension at 1× g (‘capsule enriched’), cells taken immediately after mechanical disruption of spleen tissue into single cell suspension (‘disrupted spleen’), and cells of the single cell suspension following removal of the ‘capsule enriched’ fraction and lysis of erythrocytes (‘disrupted spleen (rbc lysed)’). (b) qRT-PCR and pcdh18 qRT-PCR primer efficiency. Spleen or bone marrow cells were harvested, enriched for either CD4+ or CD8+ T cells by negative selection as described above followed by staining with CD8, CD44, CD62L, and CD127 Ab. In some experiments other cell types (fractionated spleen preparations, CD4+ or CD8+ TIL isolated from MCA 38 tumors, total spleen CD8+ T cells, bone marrow-derived dendritic cells, or spleen NK cells) were analyzed after magnetic immunobead and/or FACS purification. (c) pcdh18 is induced by LPS treatment of bone marrow-derived DC. DC were prepared from bone marrow of C57BL/6 mice by standard methods [28] and either activated by overnight treatment with LPS or not before RNA isolation and analysis. (TIF) [file pone.0036101.s001.tif]

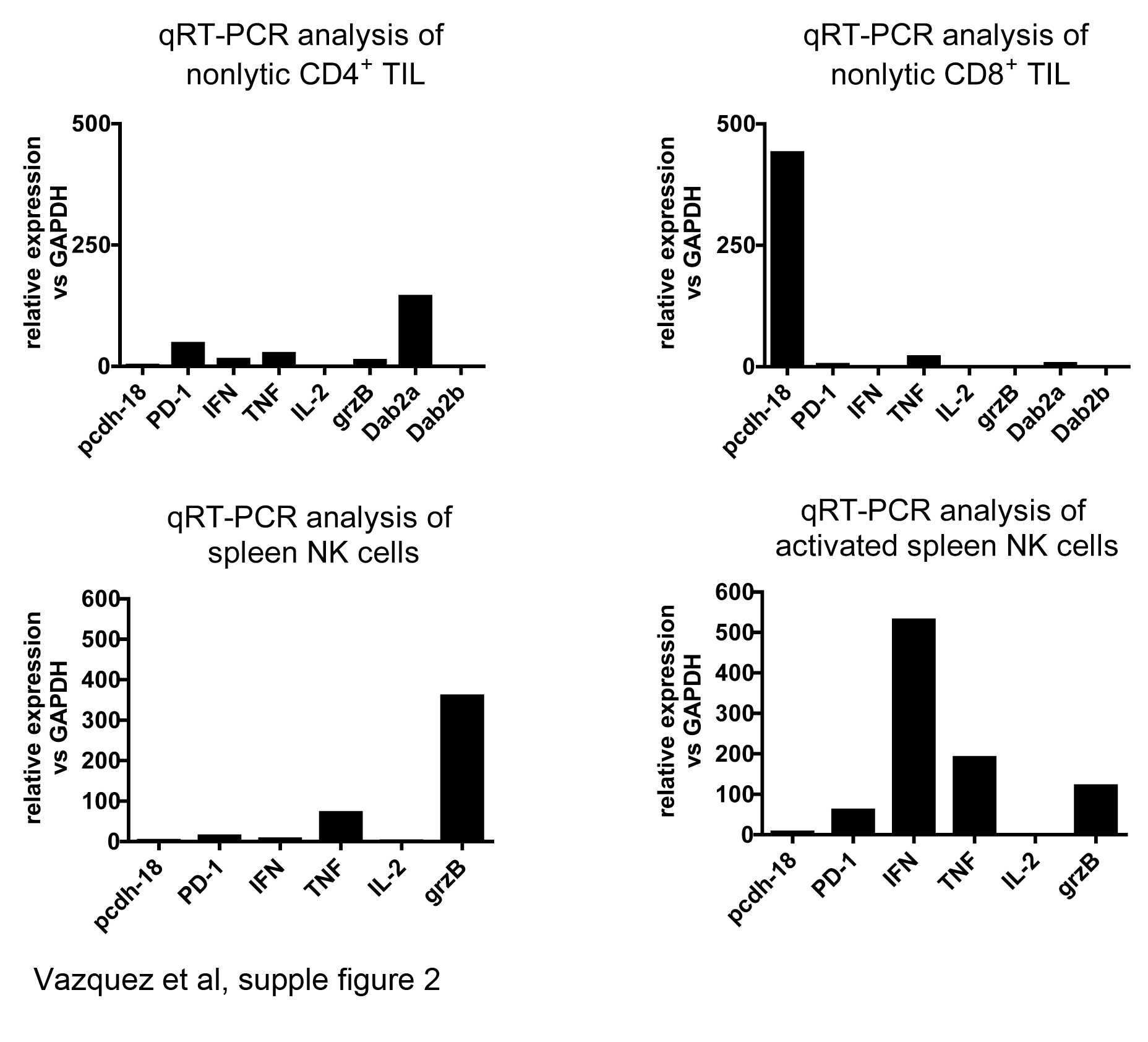

Supplement: Figure S2 — TIL qRT-PCR analyses, related to Figure 2 . Single cell suspensions of MCA38 tumors were prepared and CD4+ or CD8+ TIL were isolated by magnetic immunobeading. All data are from freshly-isolated, nonlytic TIL. NK cells were isolated by magnetic immunobead positive selection from spleens of control or mice treated 24 h prior with Poly I:C as indicated. TIL were labeled with anti-CD4 or CD8 Ab and further purified by FACS (example of flow cytometry analysis shown in Figure 2) before RNA isolation and qRT-PCR analysis. Data shown are from a single experiment of two. (TIF) [file pone.0036101.s002.tif]

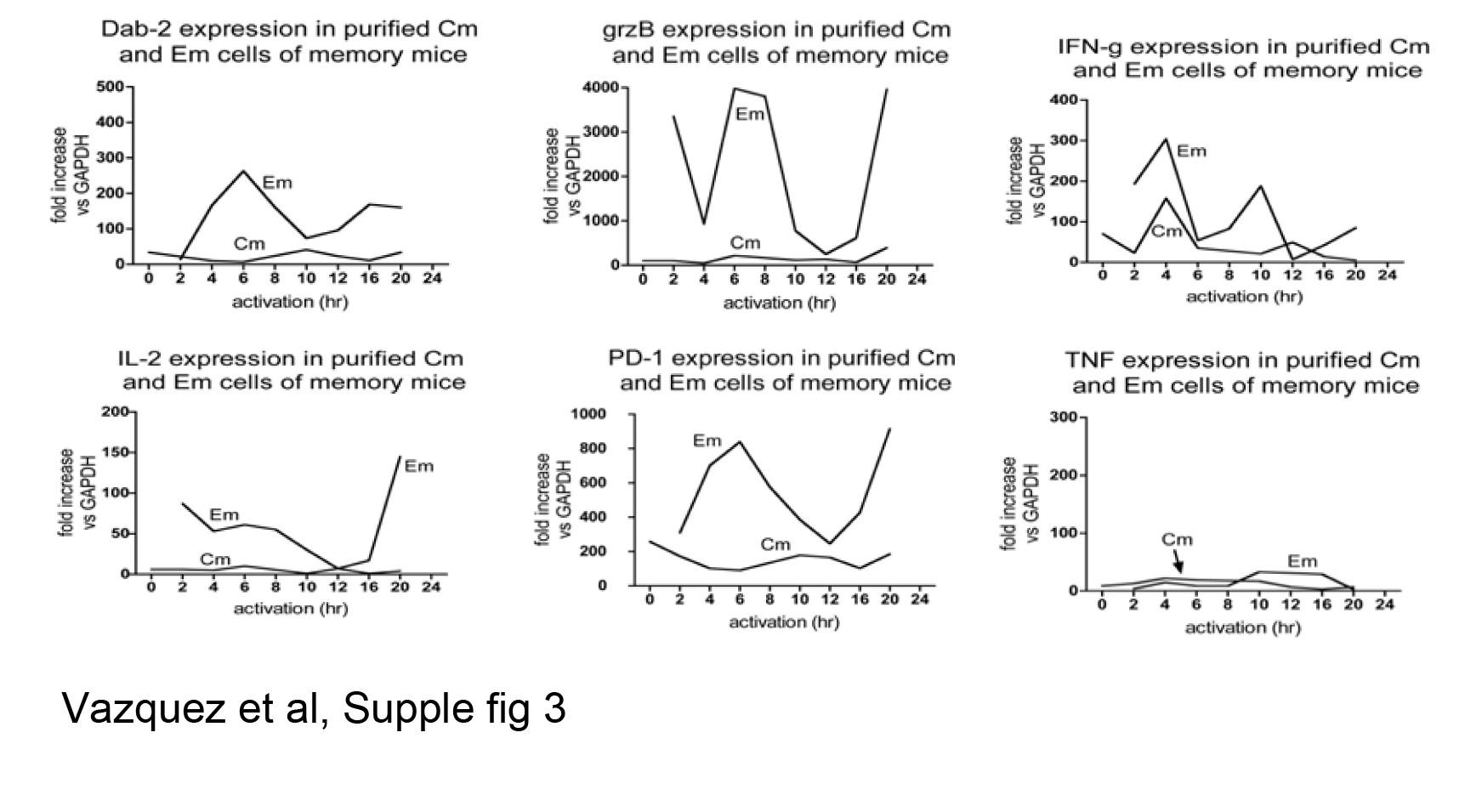

Supplement: Figure S3 — qRT-PCR analyses of FACS-purified Cm and Em cells from young, aged, or ‘memory’ mice, related to Figure 3 . Central memory cells were isolated by FACS from young (5 week), old (1 year), or memory (infected with Listeria monocytogenes at 7–8 weeks of age and recovery until 1 year old) mice. Cells were activated in vitro for the indicated times and then purified by FACS into CD62Lhi (‘Cm’) or CD62Llo (‘Em’) cell populations which provided RNA for qRT-PCR analyses. (TIF) [file pone.0036101.s003.tif]

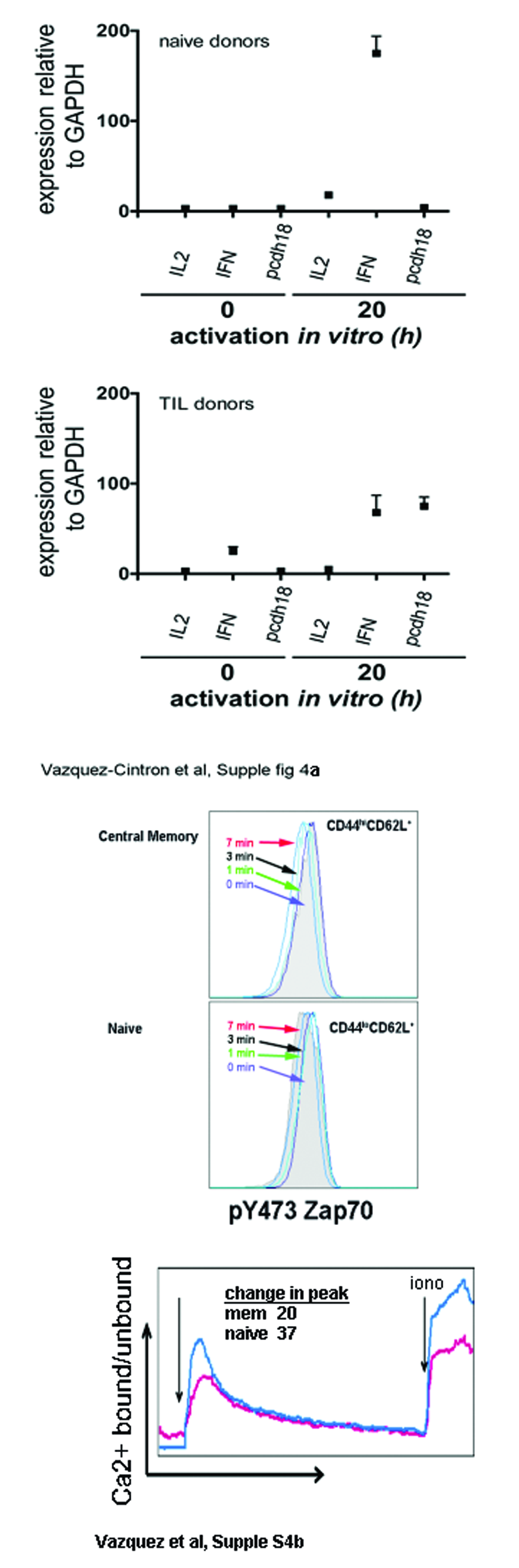

Supplement: Figure S4 — (a) AT of TIL into RAG mice, related to Figure 4. CD8+ T cells were isolated by FACS from either pooled seven week old control mice spleens or as TIL from 7 week old mice bearing MCA38 tumors for two weeks. 2×10^6^ purified cells were adoptively transferred to RAG−/− mice (n = 6). Three months after transfer, spleen CD8+ T cells were isolated by magnetic immunobeading (∼2×10^6^ cells per mouse) and analyzed by RT-PCR before or after activation in vitro (0.005 mg/mL ConA for 20 h) as indicated. (b) Analysis of proximal TCR signaling in CD8+ Cm and naive effector cells of aged mice. Spleen cells were isolated from an aged control mouse, activated with either anti-CD3/CD8 crosslinking for different times (for calcium flux) or anti-CD3e (for Zap70 analysis), then assayed for activation as described in ‘Methods’. (TIF) [file pone.0036101.s004.tif]

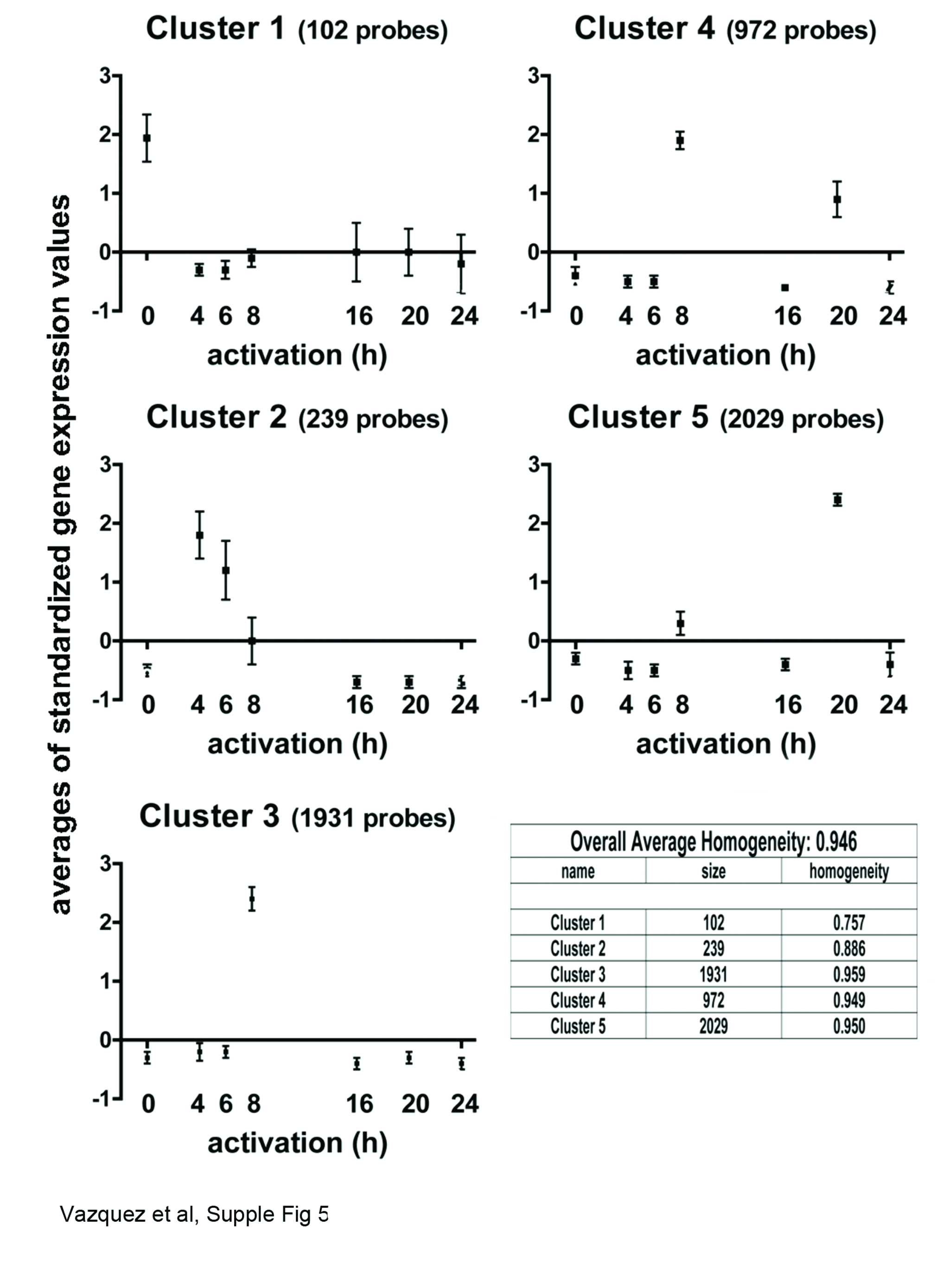

Supplement: Figure S5 — Microarray analysis of Cm cells, related to Figure 5 . Purified Cm cells were activated in vitro with Con A for the indicated times before cells were taken for RNA isolation and gene array analysis as described in ‘Material and Methods’. To obtain a subset of variably expressed genes, we calculated the coefficient of variation (CV) for each of the “valid genes” (see Material and Methods) and generated a set of 5274 “active genes” containing the transcripts with the highest (15% of the total) CV scores. For discovering prominent expression patterns we used the EXPANDER program [25] and executed CLICK, a novel clustering algorithm [26] that makes no prior assumptions on the structure or the number of the clusters. CLICK discovered five unique expression patterns. (TIF) [file pone.0036101.s005.tif]

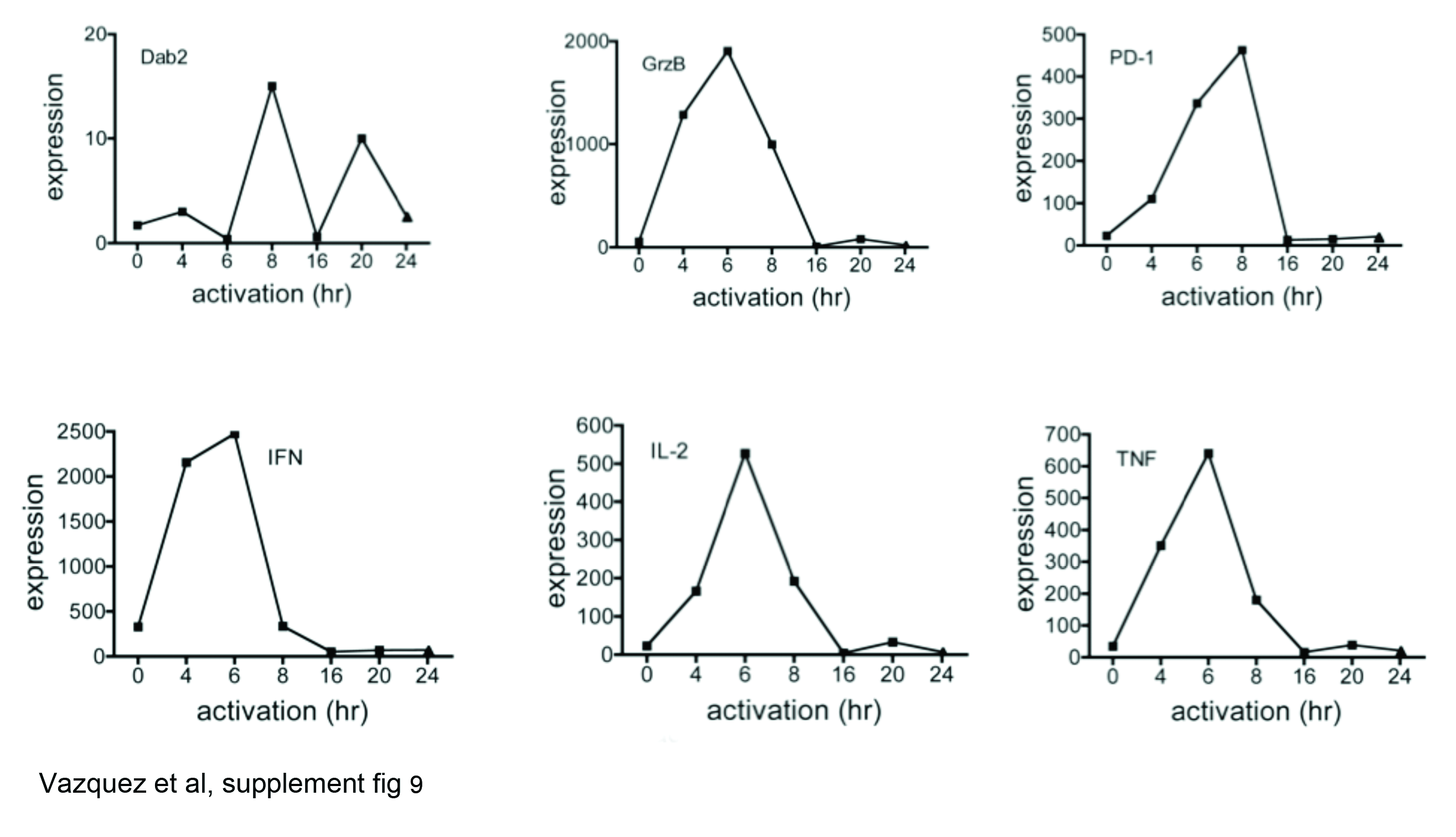

Supplement: Figure S9 — Gene array expression data for selected genes expressed in Cm cells upon activation. Relative expression at different times of activation of selected genes for which PCR validation is available (In figure S3) is shown. (TIF) [file pone.0036101.s009.tif]

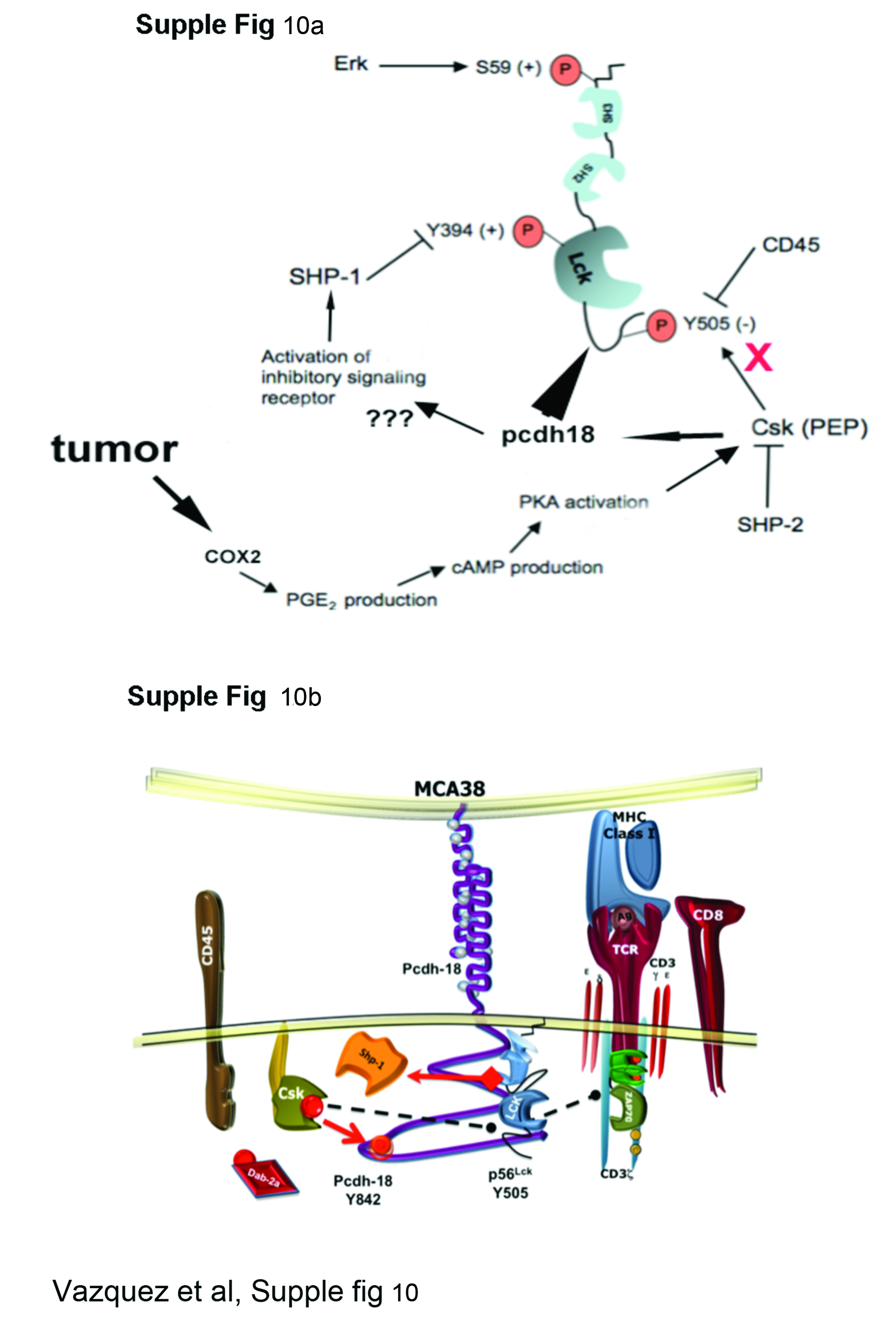

Supplement: Figure S10 — Schematic of p56lck regulation involving pcdh18, related to Figure 1 . This updated model differs from that shown in Figure1b in accommodating our data that shows that p56lck Y505 is not phosphorylated upon activation of TIL and, likely also in memory CD8+ T cells since Zap70 is not activated in primary CD8+ T cells transfected to express pcdh18 [5]. In this model pcdh18 homophilic interactions between TIL and cognate MCA38 tumor disrupt TCR mediated signaling by binding to p56lck. Binding of p56lck to pcdh18 causes a conformational change that prevents Csk (which localizes at the immune synapse with p56lck [7]) from phosphorylating the p56lck Y505 motif and thus permits phosphorylation of the homologous motif in pcdh18 (Y842). p56lck-Y384 is then available for targeting by Shp-1 causing p56lck to deactivate as observed [5]. Thus, inactivation of p56lck results in the inability to activate ZAP70, in turn preventing propagation of proximal signaling and loss of effector phase lytic function. The second diagram shows pcdh18 expressed in nonlytic TIL (or Em cells) engaged in homophilic interaction with pcdh18 expressed in a target cell; either tumor, endothelial cell, or potentially an activated DC. We propose the testable hypothesis: as a consequence of homophilic binding, recruitment of T cell pcdh 18 into proximity with Csk permits phosphorylation at Y842. Y842 phosphorylation in turn either permits or enhances binding to p56lck leading to inactivation of kinase function or sequestration from its cognate targets TCRz or Zap70. (TIF) [file pone.0036101.s010.tif]
